# Greening the Urban Landscape: Assessing the Impact of Tree-Planting Initiatives and Climate Influences on Miami-Dade County’s Greenness

**DOI:** 10.3390/rs16010157

**Published:** 2023-12-30

**Authors:** Julius R. Dewald, Jane Southworth, Jose Szapocznik, Joanna L. Lombard, Scott C. Brown

**Affiliations:** 1 Department of Public Health Sciences, University of Miami Miller School of Medicine, 1120 NW 14th Street, Soffer Clinical Research Center Room 1065, Miami, FL 33136, USA; 2 Department of Geography, University of Florida, Gainesville, FL 32611, USA; 3 School of Architecture, University of Miami, Miami, FL 33136, USA

**Keywords:** Miami-Dade County, Normalized Difference Vegetation Index (NDVI), urban greenness, spatial and seasonal analysis, climate variability

## Abstract

In urban settings, trees and greenery play a vital role in environmental well-being and community vitality. This study explores the impact of Miami-Dade County’s tree-planting initiative on urban greenness and considers the influence of climate dynamics. Using Landsat data from 2006 to 2019, we find stable overall greenness, with 5.64% of the Census blocks exhibiting significant changes. Seasonal analysis reveals winter as prominent, with 61.47% of Census blocks showing increased greenness. Temperature and precipitation, especially post-2010, correlate with greenness changes. Despite a reported increase in tree cover from 14% to 20%, our findings show only 5–6% of Census blocks with statistically significant changes, highlighting the complexity of achieving substantial improvements in green canopy coverage. The study raises questions about the efficacy of large-scale tree-planting initiatives in densely urbanized areas when human factors are not well understood. Implications for urban planning stress the importance of preserving green spaces and informed decision-making for enhancing vegetation cover in Miami-Dade County, emphasizing the need to consider local conditions, seasonal variations, policies, and human factors in urban greening efforts.

## 1. Introduction

The importance of trees and vegetation in urban areas cannot be overstated. They filter the air we breathe, provide shade to reduce the urban heat island effect, and are associated with positive mental and physical health [1–7]. The presence of trees can also contribute to a calming and restorative environment, reduced stress, and overall well-being [5–10]. Increased vegetation in urban areas can encourage physical activity by providing shade for outdoor activities such as walking, jogging, and biking, especially in warm climates, which further promotes a healthy lifestyle and reduces the risks of chronic diseases [11]. As cities across the globe have launched tree-planting initiatives, such as the Million Trees programs in Belfast, Los Angeles, Miami, and New York City and the EU commitment to plant at least 30 billion trees by 2030, it is important to assess the impacts of such initiatives in increasing greenness.

Numerous studies have explored the relationship between greenness and human health. Prior research has demonstrated that higher levels of vegetative greenness are linked to a lower BMI, decreased risk of being overweight or obese, reduced cardiometabolic conditions, and lower incidence of cardiovascular disease [12–15]. In our own work, we have linked higher levels of greenness, compared to those living in areas with lower mean greenness, at a very localized level, specifically the Census block where the individual resides, to lower risk for diabetes, acute myocardial infarction, ischemic heart disease, heart failure, and atrial fibrillation, as well as Alzheimer’s disease and depression [8,10,15]. In light of these compelling findings, fostering higher levels of greenness through tree-planting initiatives emerges as a crucial strategy not only for environmental conservation but also for promoting public health and well-being.

Developed by the Miami-Dade Community Image Advisory Board and launched in April 2011, the so-called “Million Trees Miami” campaign sought to achieve a 30% tree canopy cover for Miami-Dade County. The initial goal was based on the belief that “a healthy and sustainable urban forest provides significant social, economic, and environmental benefits to communities” [16]. Despite it not yet reaching the one million trees goal, increases in the tree cover of Miami-Dade County have been reported to increase from 14% to 20% [17,18]. As the campaign advanced through the Neat Streets Miami program into an independent Million Trees Miami initiative under the auspices of the Miami-Dade Parks, Recreation and Open Spaces department, goals have broadened to include restoration of the deteriorating urban forest in Miami-Dade County by addressing the impacts of hurricanes, development, and tree removal [19]. The primary objective of this study is to assess the effectiveness of the Miami-Dade tree-planting initiative in increasing urban canopy by conducting an analysis of the changes in greenness across Miami-Dade County. Secondarily, the paper seeks to explore how environmental factors such as rainfall and temperature may have contributed to these changes.

Previous investigations into changes over time in vegetative greenness in urban and suburban areas have relied largely on remotely sensed imagery, which can be collected frequently and with consistent methods across multiple time periods, allowing for comparisons over time [20–26]. Remote sensing data, particularly using the Normalized Difference Vegetation Index (NDVI), offer valuable insights into vegetation density and greenness by analyzing spectral reflectance. Validity studies of NDVI have demonstrated that mean NDVI at the neighborhood level has been shown to be an accurate measure of neighborhood greenness [27–29], with studies in large urban/metropolitan areas suggesting that the highest mean NDVI values are associated with parks and urban canopy and the lowest with commercial and industrial uses [28,29].

Overall, urban greenness studies using remotely sensed imagery have become more common and are increasingly important, especially considering the ongoing global trend of urbanization [30–32]. Previous studies examining NDVI in urban environments through remote sensing imagery have consistently shown its sensitivity to different vegetation types and their abundance [31]. Additionally, researchers have highlighted that mean NDVI values exhibit higher sensitivity to tree canopies and shrubs compared to grass coverage [33]. Moreover, these studies have established NDVI as a reliable metric for tracking trends in urban green space changes, provided there is a significant temporal dataset available [33]. To assess the impact of the Million Trees Miami Initiative on increasing NDVI in Miami-Dade County, this study examined changes in greenness from 2006, 5 years before tree planting began, to 2019, 8 years after the bulk of tree planting was completed.

To fully assess change in greenness relative to planting and annual growth, it is important to consider the context of Miami-Dade County’s warm and tropical to subtropical climate, which is characterized by hot and rainy summers, followed by warm and dry winters. According to the 2021 USDA Plant Hardiness Zone Map, Miami falls within zone 10b. This particular zone corresponds to an average annual minimum temperature range of approximately 1.7–4.4 °C (https://planthardiness.ars.usda.gov/, accessed on 15 September 2023). Many native and exotic plants in Miami’s urban and suburban areas are tropical or subtropical and thus are not cold-tolerant [34,35]. To determine the role of climate, we consider the role that precipitation and minimum winter temperatures may have played in changes in Miami-Dade County’s vegetation cover over time. The precipitation amount is key to vegetation growth, with decreased precipitation leading to decreased vegetation cover, while increased precipitation can promote growth [36]. As such, it is critical to identify the impact of changing climatic conditions on vegetation to attempt to separate the impact of Miami-Dade County’s tree-planting initiative from the possible effects of climate on changes in greenness.

This study aims to achieve its objectives through the examination of the temporal evolution of greenness in Miami-Dade County by analyzing NDVI scores computed from Landsat imagery. Landsat data were selected for this study because it is collected every 16 days, is available for the entire period of study, and has a 30 × 30 m resolution. We collected data beginning in 2006, 5 years prior to the launch of Million Trees Miami in 2011, to establish baseline conditions. Data were obtained through 2019, 8 years after the completion of the intensive tree-planting phase, to allow for analysis of medium-term impact. While the project was named “Million Trees”, the actual number of trees planted, to date, is expected to be substantially lower. Three of the authors (SCB, JL, and JS) reviewed the website for the initiative in 2016 which at the time provided a number below 250,000 trees planted. No number of planted trees is given on the website in 2023 (https://www.miamidade.gov/global/recreation/milliontrees/home.page, accessed on 17 September 2023).

Based on the period of time of our study analysis, we anticipate that greenness will exhibit an upward trend over time, influenced by both the maturation of existing trees and active tree planting efforts. In an effort to distinguish between the impact of tree planting and changes in climatic conditions over time, our secondary aim is to investigate the relationship between observed variations in greenness and seasonal climatic fluctuations in minimum winter temperatures and precipitation.

## 2. Methods

### 2.1. Unit of Analysis

Drawing on a Landsat dataset from a larger study (NHLBI Grant 5R01HL148880), our analysis was conducted at the level of the Census block, which provides a fine-grained understanding of how localized variations in vegetation and environmental conditions impact the overall urban landscape. Census blocks, delineated by the U.S. Census Bureau every 10 years, are statistical areas defined by visible and nonvisible boundaries, including roads, streams, and administrative divisions. They serve as the foundational building blocks for all geographic boundaries tabulated by the Census Bureau, ranging from city-like blocks in urban areas to large and irregular blocks in suburban and rural regions, providing comprehensive, wall-to-wall coverage across the United States and territories for collecting basic demographic data [37].

As noted, our own research has substantiated the considerable significance of greenness at the Census block on the incidence and/or prevalence of 13 different chronic diseases cross-sectionally and 8 longitudinally. Through cross-sectional analysis, prior research has established that Medicare beneficiaries living in Census blocks that are one standard deviation above the mean for greenness (as compared to blocks one standard deviation below the mean in greenness) experienced significant reductions in various chronic disease rates. For instance, rates of diabetes were lowered by 14%, hypertension by 13%, hyperlipidemia by 10%, Alzheimer’s disease by 18%, and depression by 28% [8,10,38]. This underscores the relevance and utility of Census blocks as the chosen unit of analysis, as it allows us to capture nuanced variations in greenness that are pertinent to important health outcomes.

### 2.2. Study Area and Data Sources

The focus of this research is to assess the changes in greenness in Miami-Dade County, which includes all Census blocks located to the east of the Urban Development Boundary (UDB). The UDB of Miami-Dade County was established to restrict development from expansion into agricultural and environmentally sensitive lands. The area east of this boundary, obtained from Miami-Dade’s open data website (https://gis-mdc.opendata.arcgis.com/, accessed on 20 July 2023), defines the geographic area available for urban development during the study period.

**Figure 1.**
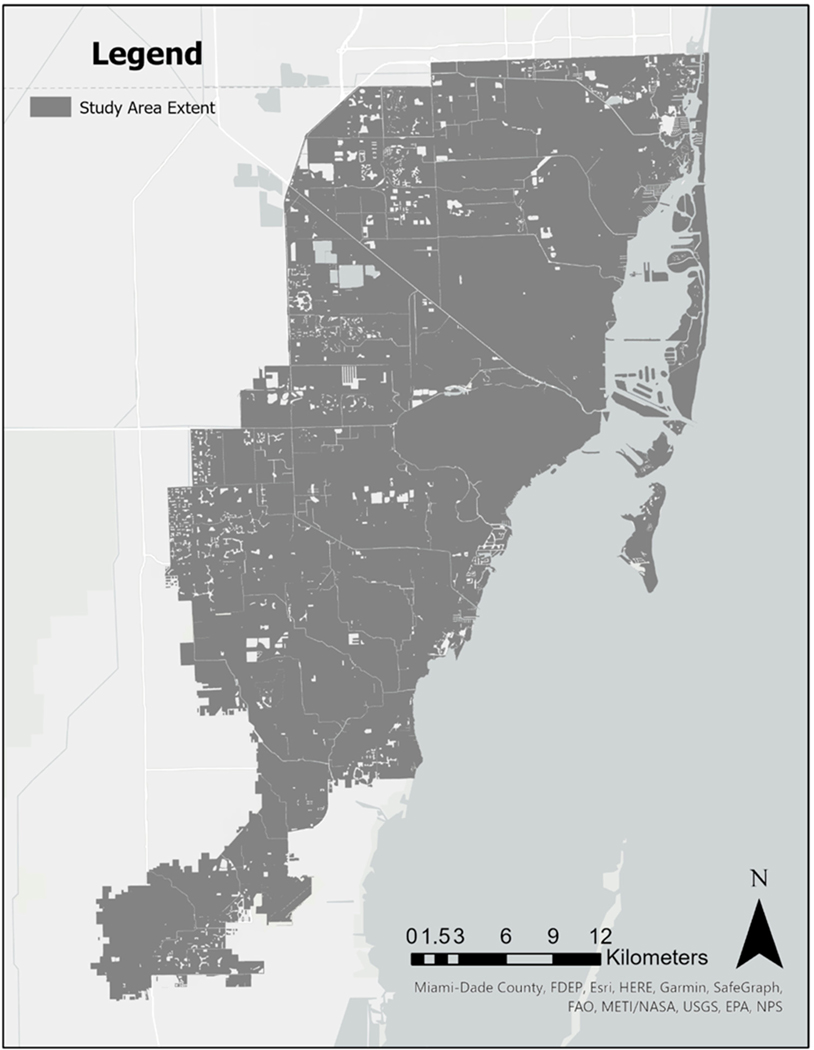
Miami-Dade County, FL, highlighting the area to the east of the Urban Development Boundary.

By deliberately excluding the Everglades and agricultural areas from the study, we can focus this study solely on vegetation within the developed areas of Miami-Dade County. This strategic focus aligns with our interest in human health, as these urban and suburban zones are where people predominantly reside. The total number of Census blocks included to the east of the UDB was 34,123. For enhanced visual clarity, refer to Figure 1 that delineates the study area in Miami-Dade County.

NDVI is an index that is used to assess the density of healthy green vegetation in a given geographical area. It is derived using remotely sensed data, typically from satellites, which measure the amount of reflected and absorbed light from the Earth’s surface. Green vegetation absorbs red light and reflects near-infrared (NIR) light. NDVI is the ratio of the difference between NIR and red bands, with these bands measuring the reflectance of light in these specified wavelengths, divided by the sum of the NIR and red bands. The resulting value ranges from −1 to 1, with higher values indicating a greater density of healthy vegetation. The formula is expressed as follows:

NDVI=(NIR−Red)/(NIR+Red)

We utilized all available Landsat images (Path 015 Row 042) between 2006 and 2019, resulting in 450 scenes, consisting of 81 images for Landsat 5, 244 for Landsat 7, and 125 for Landsat 8. The data were downloaded from the United States Geological Survey (USGS) website at https://espa.cr.usgs.gov/, accessed on 15 September 2023 and were from Collection #2, Level 1. Collection 2, Level 1 Landsat data include a USGS-calculated, pre-made NDVI imagery layer for each Landsat scene. We removed all areas of open water, cloud cover, and cloud shadows from each Landsat NDVI image to increase the validity (i.e., linked to greenness) of NDVI scores. Importantly, we refrained from applying any corrections between the different Landsat satellites, based on prior research demonstrating minimal discrepancies in NDVI values among Landsat 7 and Landsat 8 satellites in Miami-Dade County (${\text{R}}^{2}$ = 0.935, RMSE = 0.066) [39]. Similarly, because Landsat 5 and Landsat 7 have nearly identical red (a difference of 0.01 µm) and completely identical near-infrared band wavelengths, we refrained from applying any corrections. Other studies comparing Landsat satellites have also concluded that Landsat 7 and Landsat 8 imagery can be used as complementary data due to a high linear correlation coefficient (${\text{R}}^{2}$ > 0.96) [40].

### 2.3. Image Processing and Composite Creation

To create a detailed picture of land cover, we compiled the Landsat scenes into four seasonal composite images for each year throughout the study period. To achieve this, the meteorological demarcations of seasons were used to sort images. Meteorological seasons are based on the annual temperature cycle [41]. The winter season includes the coldest three months of the year in the Northern Hemisphere (December, January, and February), while the summer season covers the hottest three months (June, July, and August). The transition months of March, April, and May make up the spring season; and September, October, and November comprise the fall season.

Landsat images captured in the same season were aggregated using a median value operator for each pixel to ensure relatively cloud-free composite images; this process accounts for missing pixel values caused by cloud and cloud shadow interference by integrating data from multiple images to construct a unified and accurate image of Miami for that season. The seasonal composite images were combined using ArcGIS Pro software (version 3.0.3) to create a single multidimensional raster, allowing for analysis of spatiotemporal data using the “Zonal Statistics as Table” tool in ArcGIS Pro. Statistical summaries were calculated for each seasonal composite raster in each Census block, with each pixel included if its centroid fell within the corresponding Census block’s geographical boundary. An example of NDVI values for each of the four seasons can be seen in Supplementary Figure S1.

### 2.4. Mann–Kendall Tests for Greenness Trends

To identify significant changes in greenness throughout the study period, we analyzed the NDVI data over time using Mann–Kendall tests. This non-parametric test was used to detect monotonic trends in greenness over time within the study area. The initial test employed was the seasonal Mann–Kendall test (SK test) applied to the entire study area. This decision was motivated by the dataset’s inherent seasonal fluctuations, rendering the SK test more suitable than the traditional Mann–Kendall test. Unlike its conventional counterpart, the SK test assesses the Mann–Kendall trend within each season before aggregating the outcomes. This extensively utilized non-parametric trend analysis, common in vegetation studies [42–44], evaluates the presence of a consistent and monotonic temporal trend using Kendall’s correlation coefficient Tau.

To gauge the statistical significance of these trends, we utilized the Kendall R-programming package [45] to calculate the probability of encountering a random trend within each Census block. Our significance threshold was established at an alpha level of 0.05 and separate SK tests for each Census block were executed within the study area to identify any potential greenness changes at our selected unit of analysis.

We conducted standard Mann–Kendall tests in two distinctive manners. Initially, we carried out this test for the entire study area of Miami-Dade County across all seasons. Subsequently, we applied the standard Mann-Kendall test separately to each Census block across all four seasons. This dual approach enabled us to uncover greenness changes across all seasons across the study area and then the changes in greenness for each Census within the entire study area. Incorporating both the SK tests and the standard Mann–Kendall tests allowed us to reveal variations in greenness changes across the entire study duration, while also facilitating the detection of seasonal fluctuations. A full diagram outlining the general steps to obtain these results from the NDVI data can be found in Supplementary Figure S2.

### 2.5. Climatic Variables

To explore the potential impact of climatic conditions over time on greenness, we obtained daily precipitation and daily minimum temperature data from the Florida State University Climate Center (https://climatecenter.fsu.edu/, accessed on 2 August 2023), which provides meteorological data from weather stations across Florida. We utilized data from the weather station at Miami International Airport from 2006 to 2019. To capture any prolonged temperature trends, we extended our temperature dataset to encompass the 30-year period preceding the conclusion of our study, years 1989 to 2019. This extension conforms with the recommendation of the World Meteorological Organization, which advises acquiring a historical record spanning at least three decades for studies focused on climate change dynamics [46,47].

### 2.6. Lagged Effect of Precipitation on Vegetation

**Figure 2.**
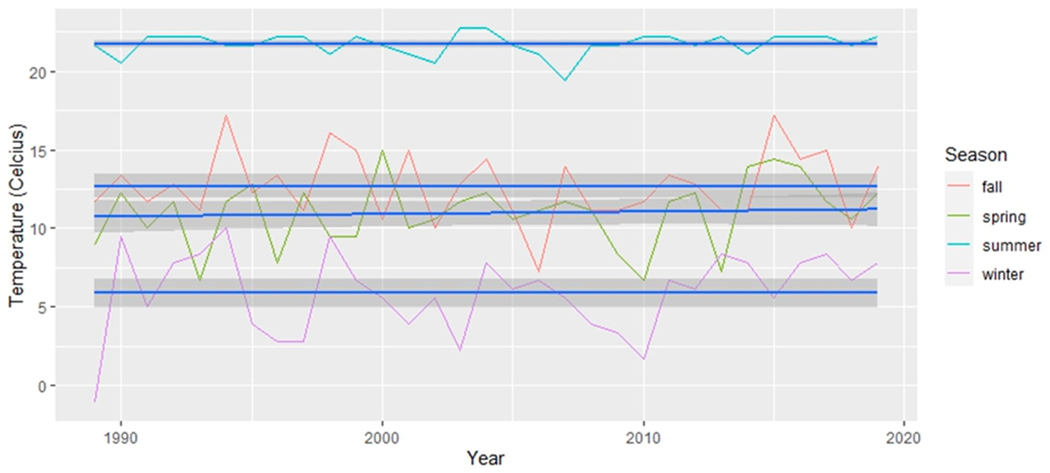
The absolute minimum temperature for each season across the 30 years (1990–2020). The line of best fit is in blue and 95% confidence intervals are displayed as dark grey areas around the line of best fit.

**Figure 3.**
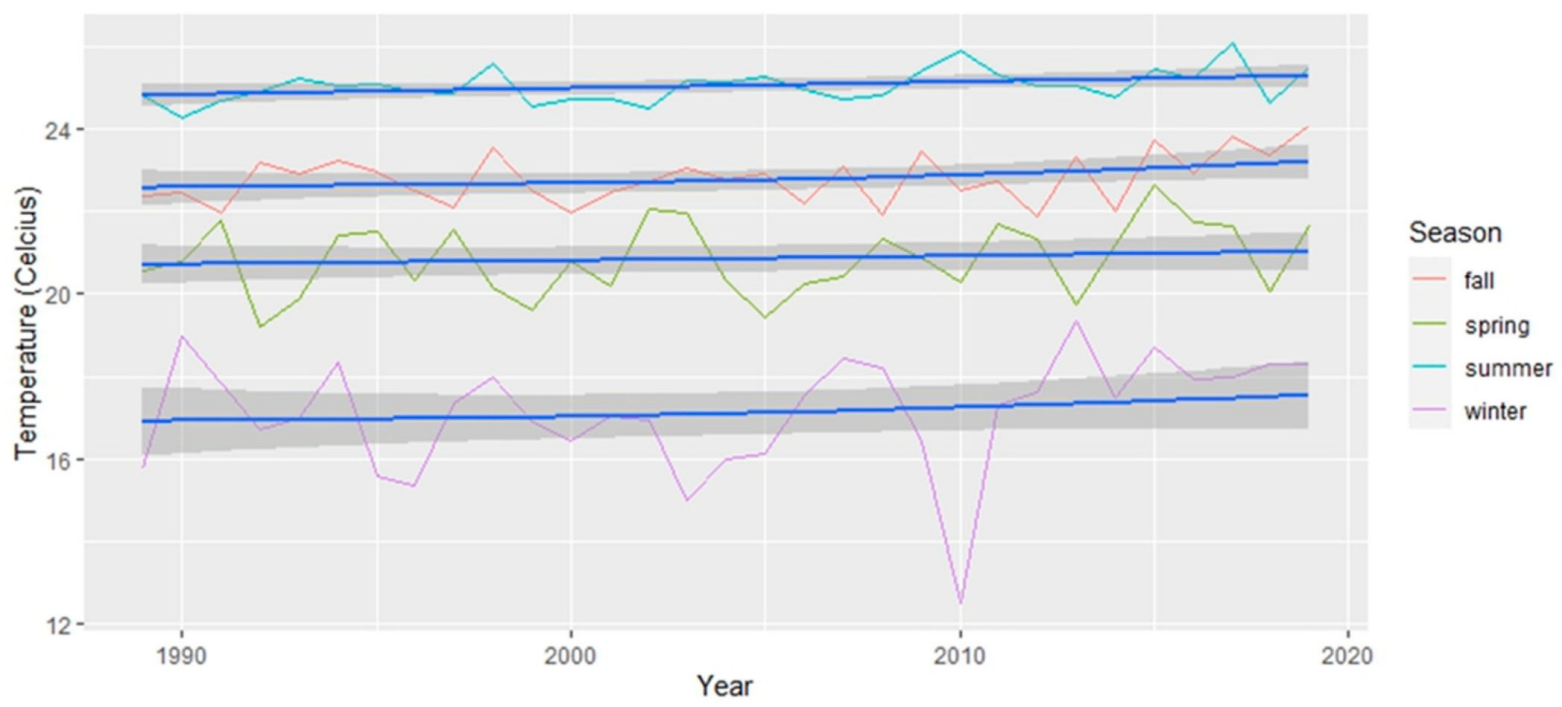
The mean minimum temperature for each season across the 30 years (1990–2020). The line of best fit is in blue and 95% confidence intervals are displayed as dark grey areas around the line of best fit.

To account for the delayed impact of vegetation’s response to precipitation, a lagged approach was applied to the monthly season categorizations of precipitation data, introducing a one-month lag, a choice supported by previous research [48,49]. Consequently, the precipitation data for winter, for example, encompassed the months of November, December, and January [50,51]. For a comprehensive analysis, we graphically presented both the absolute minimum temperature recorded during each season in Figure 2 and the average daily minimum temperature within each season in Figure 3. Subsequently, we conducted separate simple linear regression analyses on the absolute minimum temperature dataset and the average daily minimum temperature for each season. These analyses aimed to determine the presence of any significant changes throughout the study period. These analyses are particularly suitable for our aim to determine if linear relationships exist over time. The dependent variable in the GLM was the climatic variables, and the independent variable was time (in years).

## 3. Results

### 3.1. Greenness Changes in Miami-Dade County

**Figure 4.**
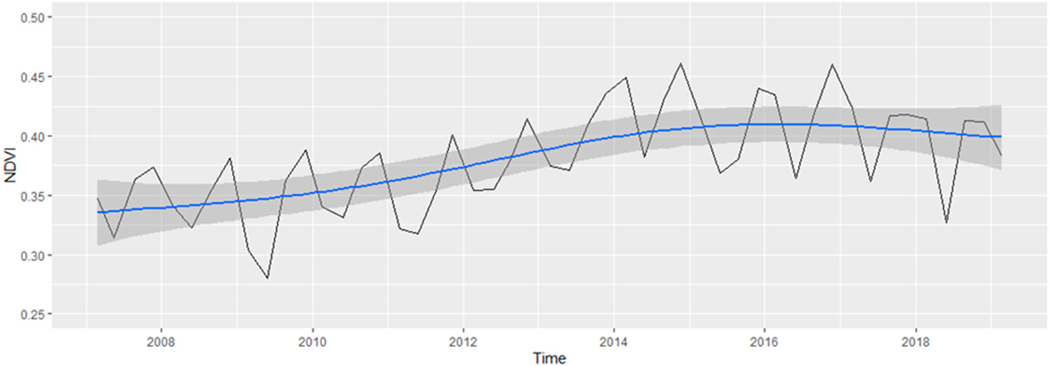
Average NDVI over time for the entire study for all of Miami-Dade County (black line). The blue line represents the line of best fit and the grey area represents the confidence interval.

**Figure 5.**
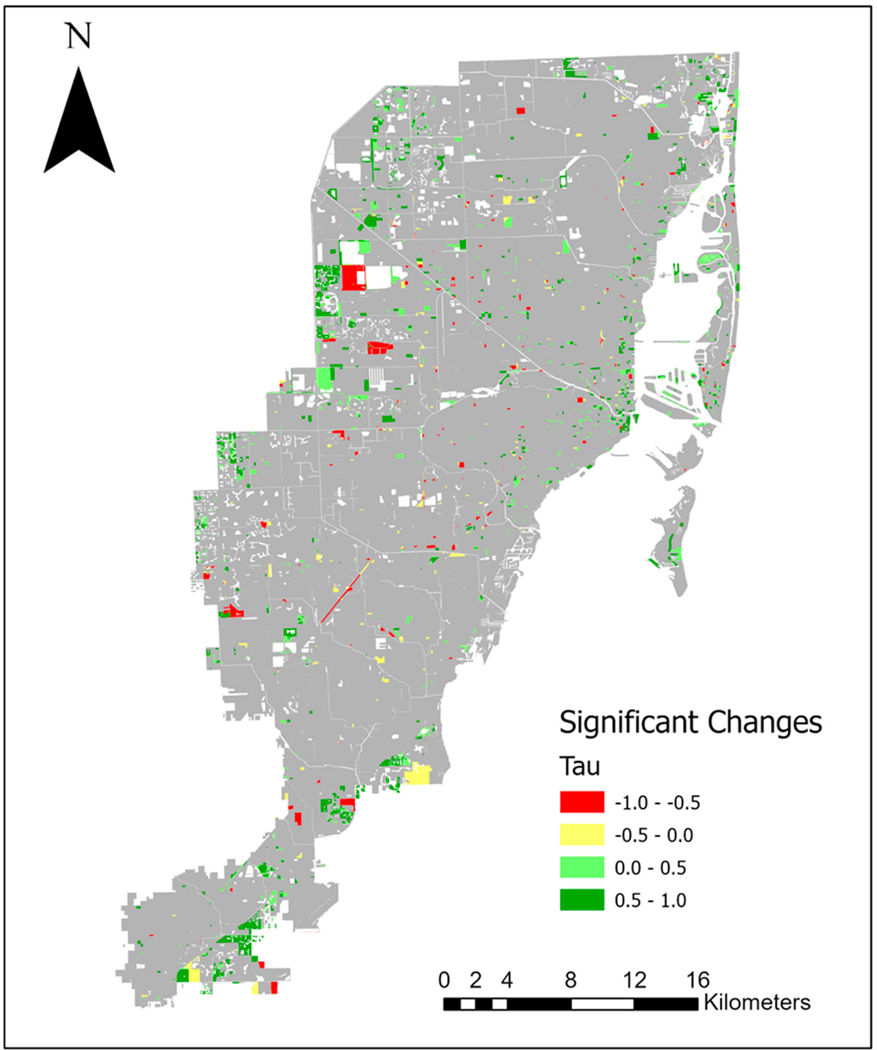
Census Blocks with significant changes in greenness from 2006 to 2019, as indicated by the seasonal Mann–Kendall test. The Tau (τ) value represents the magnitude and direction of greenness changes, with a minus sign in red areas indicating a statistically significant negative change (decrease) in greenness and a positive sign in green areas indicating a statistically significant positive change (increase) in greenness.

Figure 4 presents the NDVI score averaged across Miami-Dade County across the entire study period, and it highlights the absence of statistically significant variations in greenness (Tau = 0.026, *p*-value = 0.903). However, when examining changes in greenness at the scale of Census blocks, the SK tests revealed that 2308 blocks (6.76% of the study area) exhibited statistically significant changes out of the total 34,123 Census blocks analyzed over the 13-year period (see Figure 5). Among those Census blocks showing significant changes, roughly 83.41% displayed an increase in greenness (equivalent to 5.64% of the study area), while the remaining 16.59% experienced a decrease (equivalent to 1.12% of the study area). It is worth noting that the proportion of Census blocks exhibiting statistically significant increases at approximately 6% is very close to our significance threshold (alpha) of 0.05. Therefore, it is possible that this level of significant change in these Census blocks may be attributed to random error. This finding about the small number of Census blocks that showed significant increases at approximately 6% is consistent with the findings for the larger geographic area of Miami-Dade County for which there were no statistically significant increases in NDVI scores (i.e., greenness) across the study period. Examining the overarching pattern of greenness throughout the study period (as depicted in Figure 5), and comparing it to Figure 4, it would appear that if any NDVI increase took place, it may have occurred from 2012 to 2016, years in which tree planting was taking place. Following this period, mean NDVI values appeared to have flattened out and then slightly decreased. However, as noted, the approximately 6% of Census blocks exhibiting significant NDVI increases over the entire study period, 2006–2019, may be attributed to chance.

### 3.2. Spatial and Seasonal Patterns of Greenness Changes

**Figure 6.**
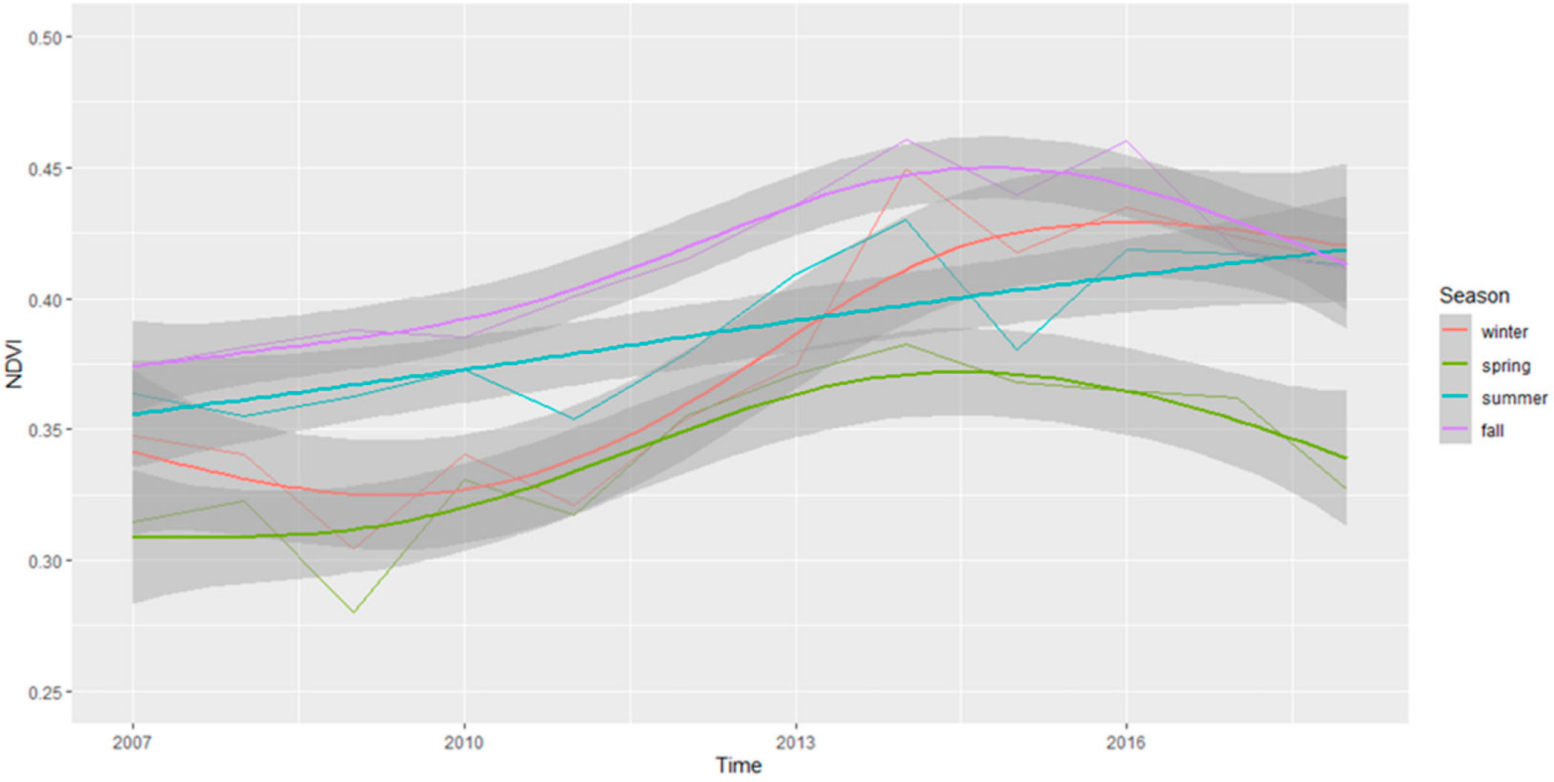
Average annual NDVI scores for each season across the entire study site of Miami-Dade County.

**Figure 7.**
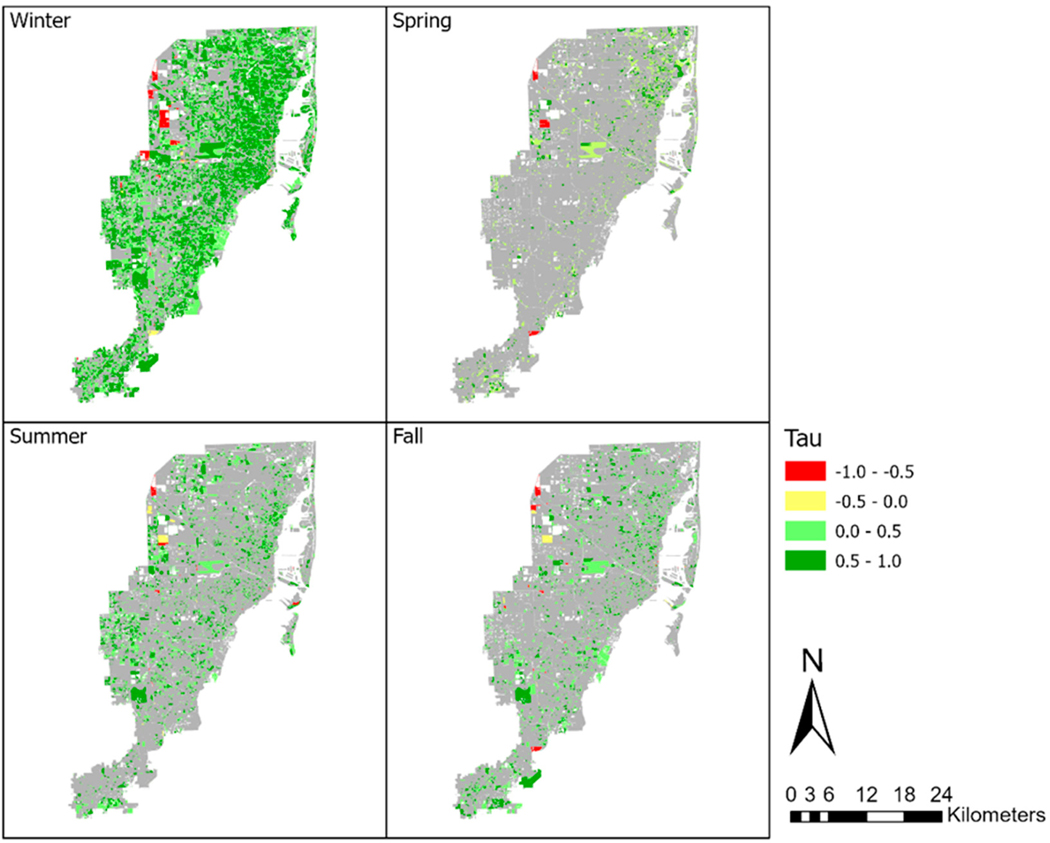
Census blocks with significant changes per season in greenness for the entire study time period (2006–2019), as indicated by the Mann–Kendall test. The Tau (τ) value represents the magnitude and direction of greenness changes, with a negative sign in red indicating a statistically significant negative change (decrease) in greenness and a positive sign in green indicating a statistically significant positive change (increase) in greenness.

We proposed a priori to conduct secondary seasonal analyses to determine how the seasons may have contributed to the overall findings presented earlier. When we explored NDVI patterns by season and examined Figure 6 over time, we observed that fall appears to show the highest NDVI values across the years, followed by summer and winter. In contrast, spring appeared to exhibit lower NDVI values. While these values do fluctuate over the study period, Figure 6 suggests that there appear to be some increases in NDVI values until around 2015, followed by a possible slight decrease until 2019. Notably, summer is an exception, as its NDVI values appear to consistently rise (non-significantly) across the entire duration of the study. The results of the standard Mann–Kendall tests separately for each individual season are presented in Table 1. Additionally, the locations of Census Blocks with significant changes for each season are shown in Figure 7. When evaluating change by season, winter, summer, and fall had significant increases in greenness, but not spring. Winter had a slope of 0.009, a statistically significant Kendall’s Tau value of 0.462, and a *p*-value of 0.032. Spring had a slope of 0.005, a Kendall’s Tau value of 0.394, and a *p*-value of 0.086. Summer had a slope of 0.006, a statistically significant Kendall’s Tau value of 0.576, and a *p*-value of 0.011. Lastly, the fall season had a slope of 0.008, a statistically significant Kendall’s Tau value of 0.606, and a *p*-value of 0.007.

As represented in Figure 7 and presented in Table 1, significant changes were observed in most Census blocks only for winter, with 61.47% of Census blocks exhibiting a statistically significant increase in greenness over the study period. Conversely, the fall season displayed the least pronounced proportion of Census blocks with statistically significant increases, with only 17.55% of Census blocks revealing a statistically significant increase in greenness. Across all seasons, the vast majority of significant changes in greenness were positive (comprising more than 99% for each season), indicating that among those Census blocks with statistically significant seasonal changes, nearly all the within-season changes were in the direction of increased greenness.

### 3.3. Visual Comparison of Greenness Changes

**Figure 8.**
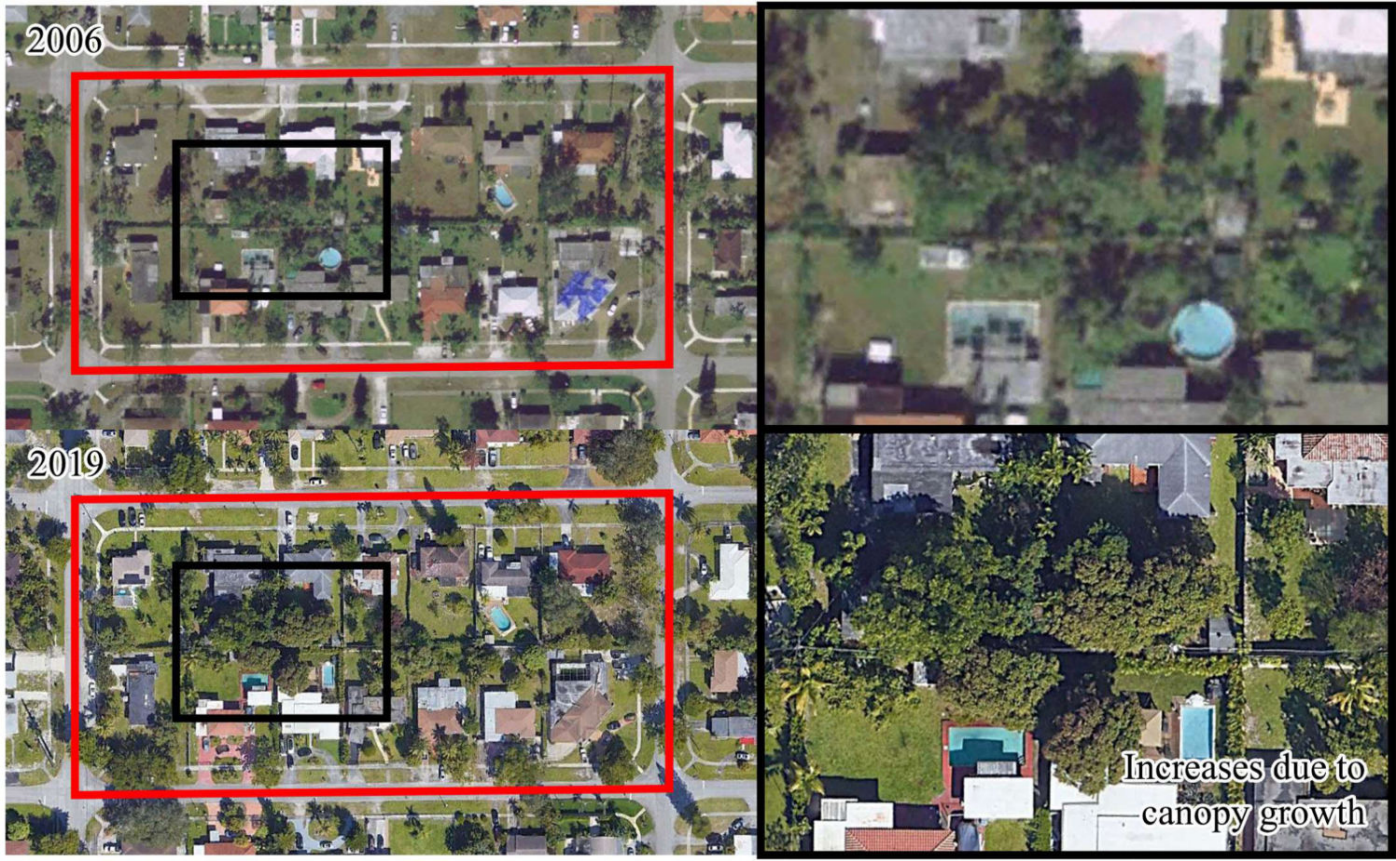
Illustration of a statistically significant increase in greenness within a Census block (in red) across the study period. The NDVI measured 0.383 in 2006 (**upper** photograph) and showed an increase to 0.483 (**lower** photograph) by 2019.

**Figure 9.**
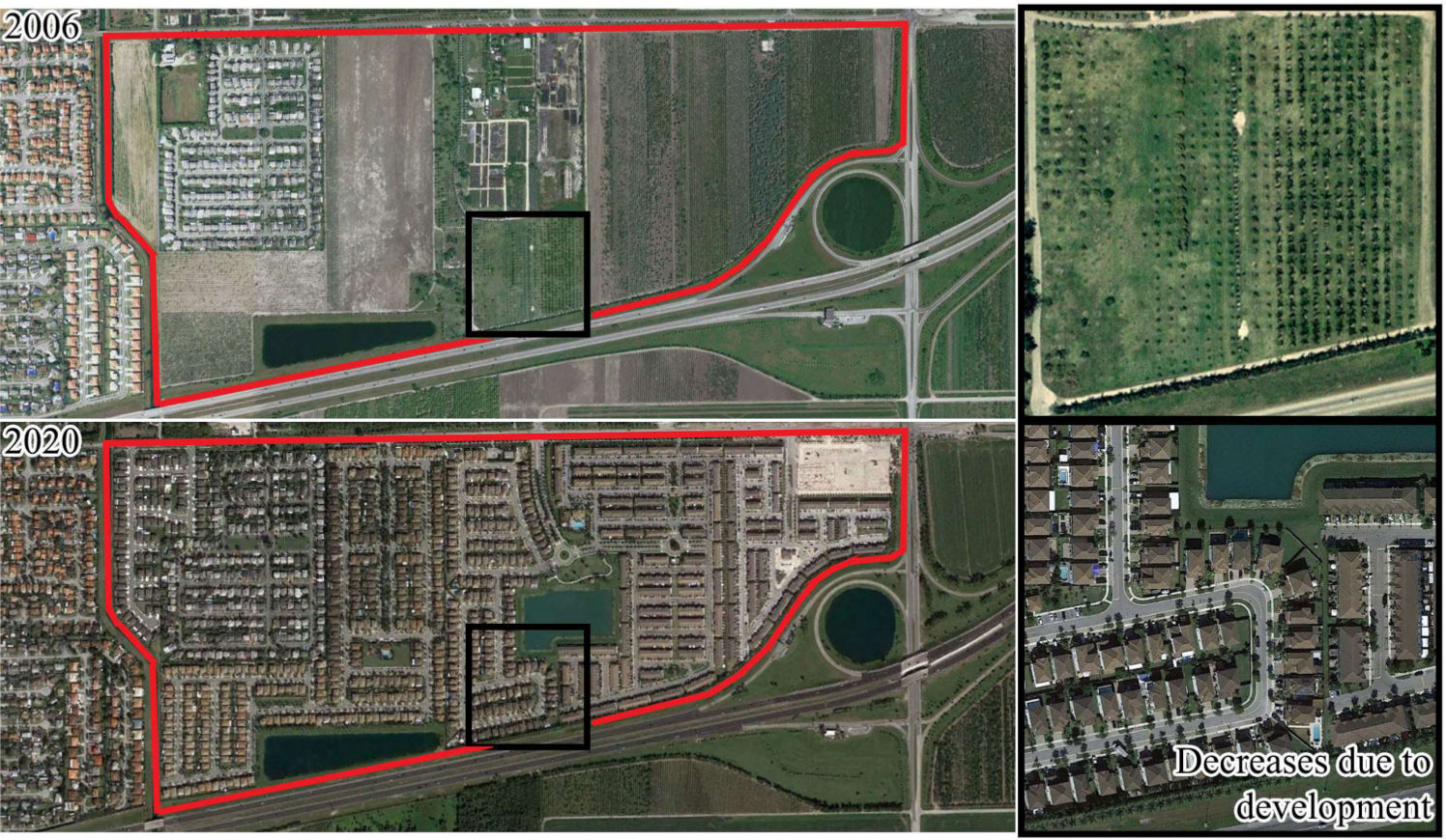
Illustration of a statistically significant decrease in greenness within a Census block (in red). The NDVI recorded a value of 0.464 (**upper** photograph) in 2006, which declined to 0.267 (**lower** photograph) by 2020. It is worth noting that due to discrepancies in image acquisition timing, the Google Earth imagery does not perfectly align with the start and end dates of the Landsat imagery due to a lack of available images for 2019 at this location.

To illustrate the changes in greenness, two locations within Miami-Dade with statistically significant changes in vegetation are presented (Figures 8 and 9). Initially, both locations had moderate NDVI values (approximately 0.50 NDVI). However, over time, one location experienced a statistically significant increase in vegetation, while the other displayed a statistically significant decrease. Using Google Earth, Figure 8 illustrates increased vegetation in one location that may be attributed to the maturation of existing trees and tree plantings. This Census block exhibited statistically significant increases across the spring, fall, and winter seasons. In contrast, Figure 9 shows a location that demonstrated statistically significant reductions in vegetation during the fall and spring seasons, mainly due to the conversion of open fields and farmlands into residential areas. Notably, discrepancies in image acquisition timing between Google Earth and Landsat imagery should be acknowledged, as the former does not precisely align with the latter’s start and end dates.

### 3.4. Climatic Variables: Minimum Temperature

**Figure 10.**
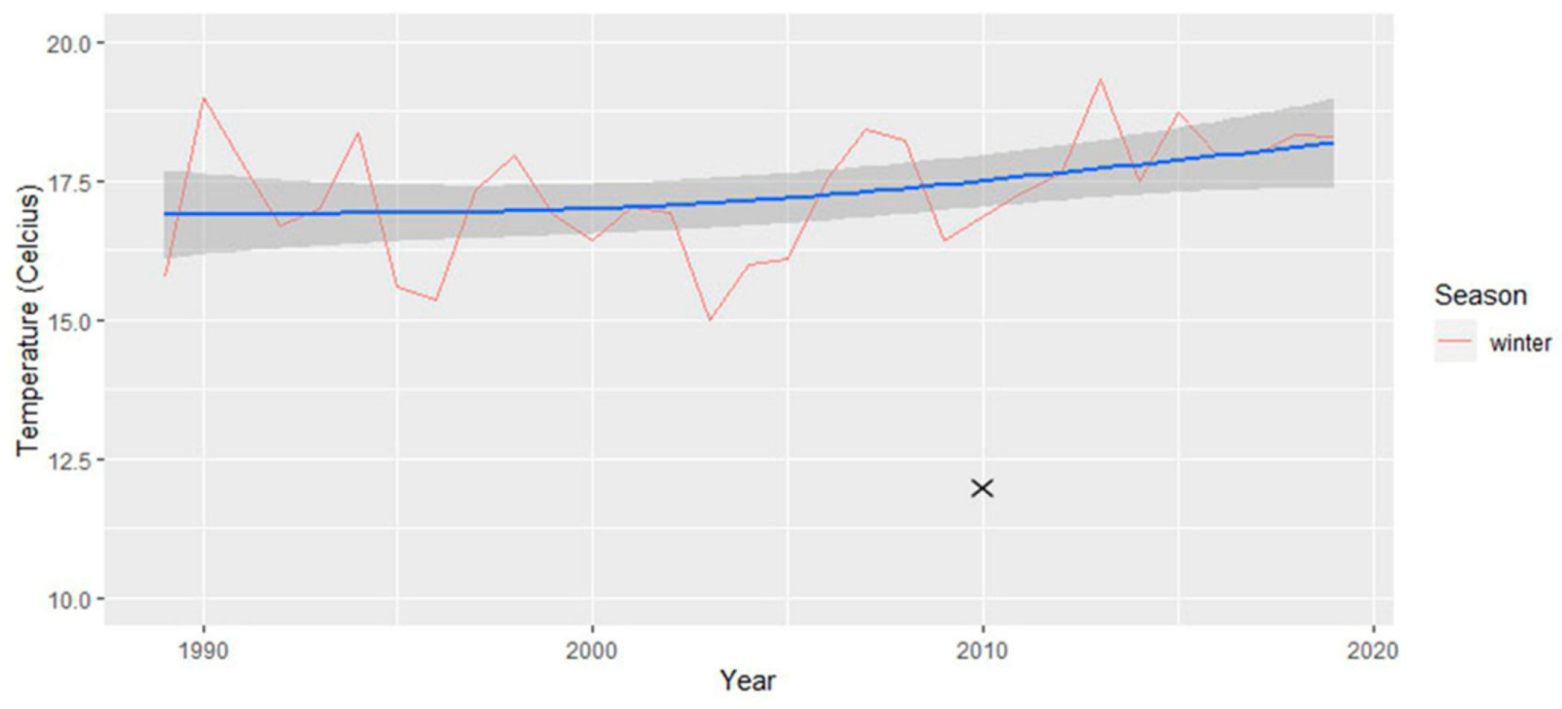
The mean minimum temperature for winter across 30 years (2020–1990). Within this graph, the 2010 outlier was removed, as indicated by the “x” marking the location of the outlier. The line of best fit is in blue and 95% confidence intervals are displayed as dark grey areas around the line of best fit.

The analysis of changes in temperature over time used generalized linear models (see Table 2). Within each season, the absolute minimum temperature (Figure 2) revealed no statistically significant changes. In contrast, the mean minimum temperature (Figure 3) revealed a statistically significant increase over time in temperature for the summer season (*p* < 0.05). Although the winter season did not exhibit a statistically significant increase in mean minimum temperature, an extremely low value was observed in 2010. The exclusion of this outlier data point from 2010 resulted in a statistically significant increase in the mean minimum temperature over time for winter, particularly in the last 10 years, as depicted in Figure 10 (generalized linear model: ${\text{R}}^{2}$ = 0.1186, *p* = 0.035).

### 3.5. Climatic Variables: Precipitation Variability

**Figure 11.**
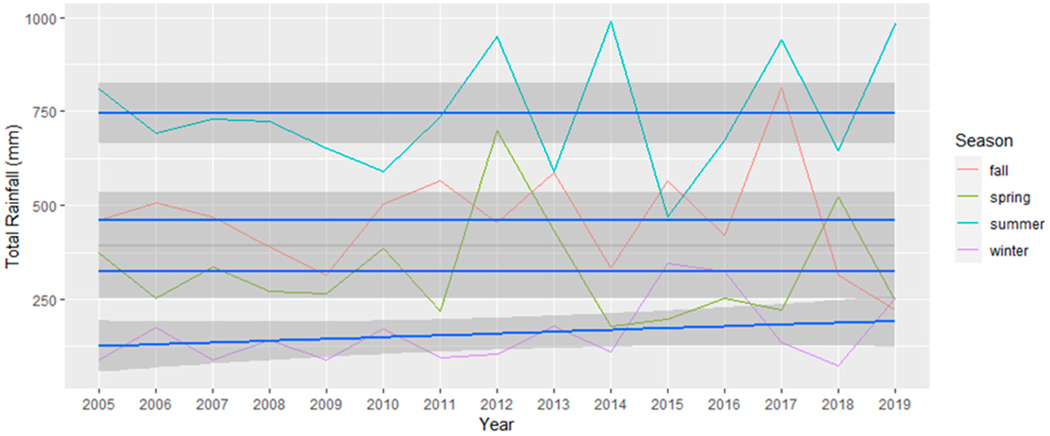
The total amount of rainfall for each season across the study period. The line of best fit is in blue and 95% confidence intervals are displayed as dark grey areas around the line of best fit.

Precipitation levels in Miami throughout the study period, presented in Figure 11 and Table 2, showed no statistically significant changes over time for any of the four seasons (winter *p*-value = 0.123, spring *p*-value = 0.893, summer *p*-value = 0.493, fall *p*-value = 0.831). Figure 11 shows that there is increasing variability in precipitation totals after 2010, found across all seasons. Sudden fluctuations in rainfall patterns, even when the overall amount of rainfall remains relatively stable, can significantly impact the productivity of vegetation and thereby the greenery within a specific region [52,53]. This effect becomes especially evident during the dry season, as an increase in rainfall during this period can lead to increased vegetation growth [54]. To assess whether this observation is statistically supported, we divided the precipitation data into two sets: pre-2010 and post-2010. We calculated the variance for each season in both time periods and conducted a Shapiro–Wilk test to evaluate normality. Because normality was found for both pre- and post-2010 samples, we proceeded with an F-test to determine if the differences in variance before and after 2010 were statistically significant.

**Figure 12.**
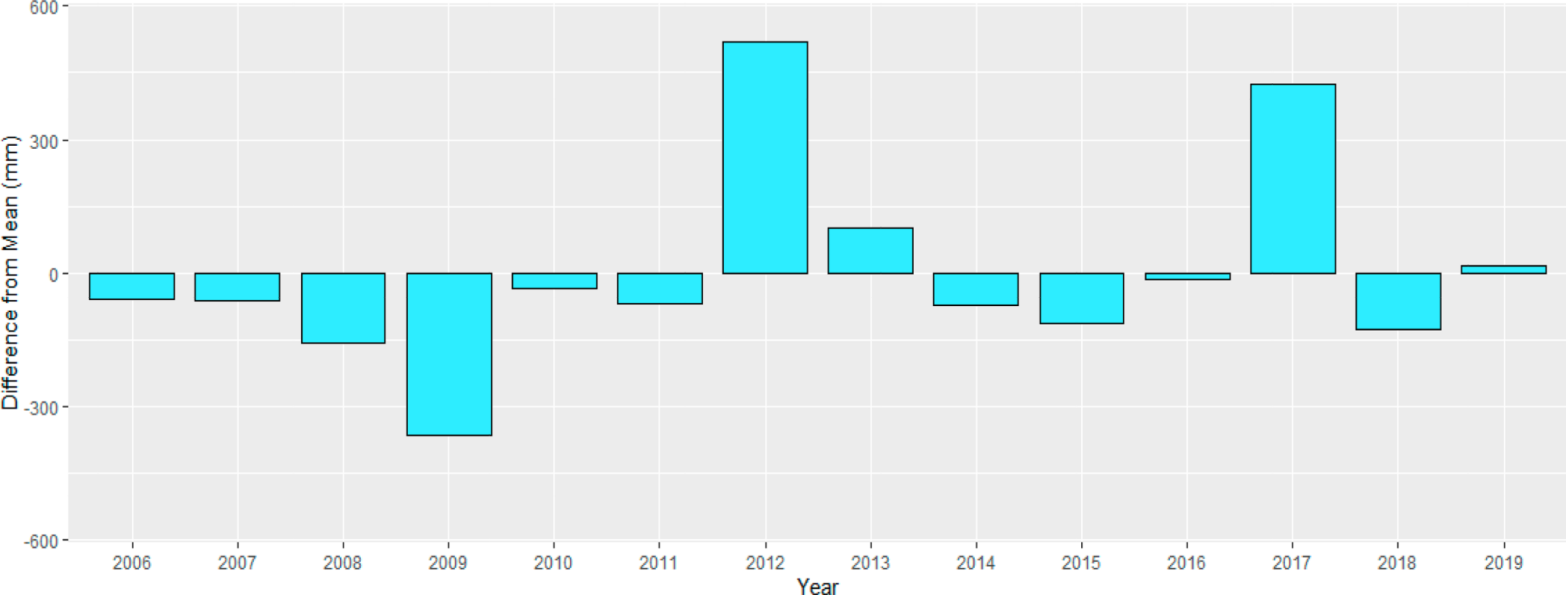
Annual deviation from the mean rainfall (1687 mm) in Miami-Dade County.

The outcomes of the F-test (see Table 3), with respect to variance equality, indicate discernible distinctions in precipitation variability within seasons across time. Specifically, for the fall and winter seasons, while the *p*-values were close to 0.05, the variance differences (pre- vs. post-2010) did not reach statistical significance (fall *p* = 0.072, winter *p* = 0.07). Conversely, for the spring and summer seasons, we observed significant variance differences (spring *p* = 0.024, summer *p* = 0.048), suggesting a statistically significant increase in precipitation variability following 2010. Furthermore, we conducted normality tests utilizing the Shapiro–Wilk method to assess data distribution. Both the fall and summer seasons exhibited normally distributed precipitation data for both the pre-2010 and post-2010 periods. In contrast, the spring season displayed a statistically significant variance difference accompanied by non-normal distribution in the post-2010 period. Similarly, the winter season exhibited a non-normal distribution in the pre-2010 period, which transitioned to a normal distribution after 2010. Finally, Figure 12 illustrates the annual deviation from the mean rainfall (1687 mm) in Miami-Dade County during the years of the study period. The deviation highlights certain years experiencing more or less rainfall than the average, with no discernible pattern in the overall amount of rainfall.

## 4. Discussion

### 4.1. Overview of Findings

The objective of this study was to investigate changes in greenness (i.e., vegetation amount/density) across Miami-Dade County over a 13-year period (2006–2019) and to evaluate how these changes align with the objectives of the Million Trees Miami project (2011–ongoing). Demonstrating a lack of impact by the tree-planting initiative on the overall greenery for the whole of Miami-Dade County, there was no statistically significant change in NDVI/greenness scores across the study period. Consistent with these overall findings, only 6.76% of the studied Census blocks displayed statistically significant changes, over 99% of which were in the direction of increased greenness. Additionally, the study aimed to ascertain whether alterations in spatial and temporal greenery patterns were associated with shifts in climate patterns. Winter, summer, and fall showed statistically significant increases in greenness, with winter having the highest proportion of Census blocks with significant changes (61.47%), over 99% of which were in the positive direction. It is important to note that Million Trees Miami is the name of the tree-planting campaign, but Miami-Dade County seems to have planted only about one-fourth of that amount as of 2016, when three of the authors (SCB, JL, and JS) reviewed the website for the initiative in 2016. No number of planted trees is given on the website in 2023.

### 4.2. Prior Greenness Studies in Miami-Dade County

Our study builds upon a foundation of prior research conducted in Miami, allowing us to contextualize and compare our findings with previous work in the area. Notably, previous studies have focused on assessing tree cover in Miami-Dade County. For instance, as of 2020, research indicated that the tree canopy in Miami-Dade County’s urban areas stood at 20.1%, notably lower than the national average of 28.8%, but higher than the initial 14% cover at the start of the Miami tree-planting initiative [17,18].

Furthermore, earlier research delved into tree cover changes from 2003 to 2009, revealing a concerning 1.7% decrease in tree cover for Miami over this 6-year period [18]. Additionally, a more recent investigation explored variations in Miami-Dade’s tree canopy from 2016 to 2020, ultimately finding no significant alteration in the overall tree canopy percentage within the county during this 4-year period [55]. These findings offer a temporal perspective on Miami’s greenery, contributing to our understanding of trends leading up to and following our study period.

In addition to these broader trends, our study delves into the spatial variation of greenery changes within Miami-Dade County. Fine-scale potential changes, such as clearing, planting, pruning, and urban developments, play a significant role in shaping the local green landscape. Our focus on spatial distribution enables us to identify hotspots, offering a more nuanced understanding of the dynamics at play within the county.

### 4.3. Extent of Changes in Greenness

The results of the seasonal Mann–Kendall test, depicted in Figure 4, indicate that approximately 6% of the total examined Census blocks in this study exhibited statistically significant increases in greenery across the entire 2006–2019 period, suggesting a relatively stable level of greenery throughout Miami-Dade County. This is consistent with the findings of the overall changes in greenery across all of Miami-Dade County during the study period. However, when we analyzed greenness changes within each of the four meteorological seasons, a notable proportion of significant changes became evident. Specifically, during the winter season, a total of 20,976 Census blocks (equivalent to 61.47% of the total) exhibited statistically significant changes in greenness (Table 1), 99.46% of which were positive indicating a statistically significant increase in greenness. The results for the remaining three seasons were also significant but at much lower rates of change, with summer having 8407 Census blocks or 24.64% of the total, spring with 6406 Census blocks or 18.77% of the total, and fall with 5990 Census blocks or 17.55% of the total having significant greenness changes. Notably, all seasons had over 99% of these changes being positive changes. These results highlight the season-specific variations in greenness changes, with winter displaying the highest proportion of statistically significant positive changes. This observation emphasizes that, within this region, winter has shown a conspicuous increase in greenness across a substantial portion of the landscape. Since these significant changes in greenness primarily occur during a single season and are not apparent in the overall landscape trends, it suggests that factors other than a general increase in canopy cover are likely responsible for these changes. If increasing canopy was the driving force, we would anticipate consistent upward trends in greenness across all seasons.

### 4.4. Million Trees Miami Initiative Evaluation

Considering the findings presented in this study, it is important to consider the role of the so-called “Million Trees Miami” planting initiative and its potential impact on the observed increase in greenness. While the reality of this initiative was about one-fourth of the intended goal of one million trees, we should still expect to see canopy coverage increase as a result of this program. Therefore, it was unexpected that our findings revealed that greenness across the whole county for the 13 years of the study period did not show statistically significant increases. In fact, across the study period, only 6% of Census blocks showed statistically significant increases in greenness—which could occur at random when using a *p*-value of <0.05. It is therefore concerning that the planting of trees in Miami-Dade County has not shown substantial improvements in greenness, even 8 years after the first trees were planted.

The current analysis covers the years 2006 to 2019, while the bulk of the planting initiative occurred between 2011 and 2016. It is therefore possible that the saplings planted during this period may not have reached a stage of maturity that significantly contributes to the observed changes in greenness. Considering the typical growth rates of trees, particularly in the context of urban environments, the saplings planted as part of the initiative are unlikely to have reached a stage where they can substantially impact the overall greenness within the studied timeframe. Many factors, including species selection, local conditions, and maintenance practices, influence the growth and establishment of newly planted trees. These saplings need time to establish their root systems, develop foliage, and contribute significantly to the overall greenness of their location. As such, while the “Million Trees Miami” project may hold the potential to significantly impact the urban canopy in the long term, the observed changes in greenness, found predominantly within the winter season only, across the study period are much more likely to be influenced by a combination of factors linked to changing dynamics of this season, such as lower overnight minimum temperatures and changes in the distribution of precipitation. If the changes in greenness were a result of increased tree canopies and tree counts, we would expect to see a much more consistent increase in greenness year-round, not just in winter. It is possible that in addition to planting trees, it may be necessary to protect mature trees more aggressively. In addition, as a counter to the tree-planting initiatives, there is still significant development in the region, such that some areas may be experiencing tree removals, tree pruning, and other human-induced reductions in the canopy. For Miami-Dade County, the results of this study demonstrate that, in the short term, planting trees is insufficient to increase greenness as measured by NDVI.

### 4.5. Limitations

These results suggest the value of monitoring vegetation changes in Miami-Dade County to assess challenges posed by urbanization, climate variability, and other human factors related to meeting urban planning goals. This study uses limited data sources and may have excluded important variables that could influence the observed results. Further research is needed to validate the findings and explore potential explanations for the observed trends. Additionally, the lack of explicit details regarding the locations or quantities of tree plantings under the Million Trees Miami project poses a challenge in directly assessing its impacts. Furthermore, the omission of an examination of vegetation types represents a notable gap in our analysis.

Our use of the Census block as the unit of analysis facilitated the assessment of greenness variability at very high resolution. In contrast, our climate data are derived from climate stations and therefore lack a comparable spatial scale. While we can discuss broad-scale climate changes for the entire region (Figures 2, 3, 10 and 11), establishing statistical linkages proves challenging due to these significantly different spatial scales. Nevertheless, as we delve into potential climatic drivers of change, such as slightly wetter winters and warmer minimum temperatures likely associated with increased greenery during the same season, it is important to note that significant spatial variation persists across the study area, highlighting other potential drivers of change, including tree planting and other human-related factors. An in-depth analysis would be required to establish potential causal relationships.

### 4.6. Conclusions and Future Research

The findings from this study offer valuable insights into the dynamics of vegetation in Miami-Dade County, especially within the context of recent climate fluctuations. These insights are crucial for assessing the impacts of urban greening initiatives and for informing future policies and urban planning efforts. The knowledge gained from this study can inform urban planning and policymaking decisions in Miami-Dade County in several ways. Strategies and interventions based on the insights obtained include the importance of preserving existing green spaces and in particular preserving canopy from mature trees, timing, and designing greening initiatives based on seasonal patterns, promoting tree preservation efforts, and incorporating green infrastructure into urban development plans. By integrating these findings into decision-making processes, Miami-Dade County can enhance its vegetation cover, promote the health and well-being of its residents, and create more sustainable and resilient urban environments.

## Supplementary Material

Supplementary Material

## Figures and Tables

**Table 1.** Number and percentage of Census blocks with significant greenness changes.

Period	Number of Census Blocks with Significant Change in Greenness	Percent of Total	% of the Significant Changes Were Positive (Tau > 0.0)	% of the Significant Changes Were Negative (Tau < 0.0)

Total	34,123	-	-	-
Winter	20,976	61.47%	99.46%	0.54%
Spring	6406	18.77%	99.47%	0.53%
Summer	8407	24.64%	99.43%	0.57%
Fall	5990	17.55%	99.02%	0.98%

**Table 2.** Linear model results for climatic data.

Variable	Season	R	R Square	Adjusted R Square	Residual Standard Error	*p*-Value

Absolute minimum temperature	Winter	0.170	0.029	−0.004	9.112	0.359
	Spring	0.224	0.050	0.018	9.012	0.225
	Summer	0.093	0.009	−0.026	9.208	0.620
	Fall	0.005	0.000	−0.034	9.247	0.981

Mean minimum temperature	Winter	0.226	0.051	0.018	9.009	0.222
	Spring	0.247	0.061	0.028	8.962	0.181
	Summer	0.436	0.190	0.163	8.321	0.014
	Fall	0.348	0.121	0.091	8.670	0.055

Rainfall	Winter	0.416	0.173	0.110	4.220	0.123
	Spring	0.038	0.001	−0.075	4.638	0.893
	Summer	0.192	0.037	−0.037	4.555	0.493
	Fall	0.060	0.004	−0.073	4.633	0.831

**Table 3.** Comparative analysis of seasonal precipitation variability in Miami: pre-2010 vs. post-2010.

Season	Variance Pre-2010	Variance Post-2010	Variance Difference	F Test *p*-Value	Shapiro–Wilks Pre-2010 *p*-Value	Shapiro–Wilks Post-2010 *p*-Value

Fall	5589.686	31,799.880	26,210.190	0.072	0.258	0.795
Spring	3402.591	32,505.830	29,103.240	0.024	0.209	0.020
Summer	5462.845	37,622.470	32,159.630	0.048	0.959	0.201
Winter	1814.336	10,429.760	8615.420	0.070	0.046	0.103

## Data Availability

The data presented in this study are openly available in Dryad data repository.
