# Supplementary material for: Greening the Urban Landscape: Assessing the Impact of Tree-Planting Initiatives and Climate Influences on Miami-Dade County’s Greenness
Source: Remote Sens (Basel). Author manuscript; Available in PMC 2025 Jan 17. (PMC11741672; doi:10.3390/rs16010157)
Supplement: Supplementary Material [file NIHMS2002736-supplement-Supplementary_Material.pdf]

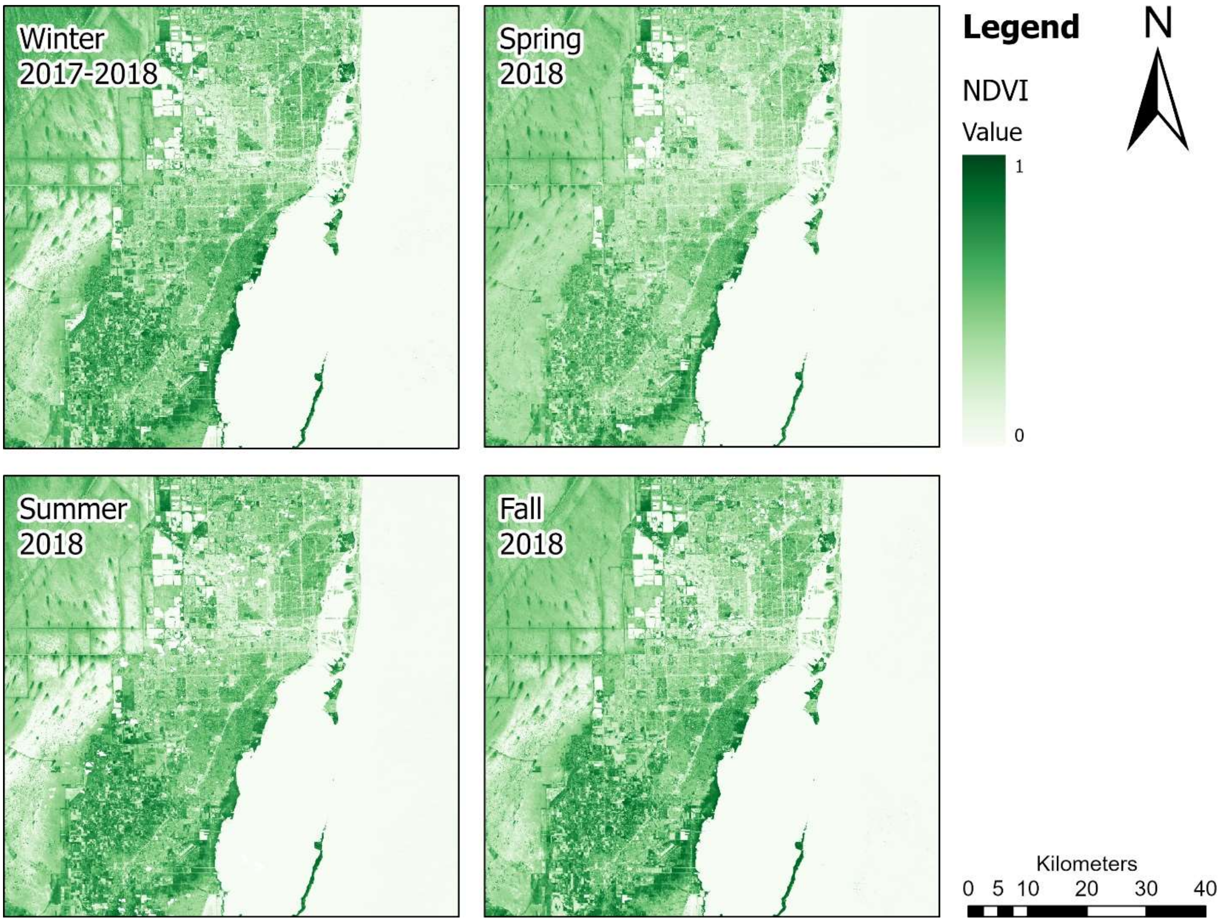

Supplementary Figure S1. Examples of composite NDVI images representing each season throughout the entire study area in 2018.

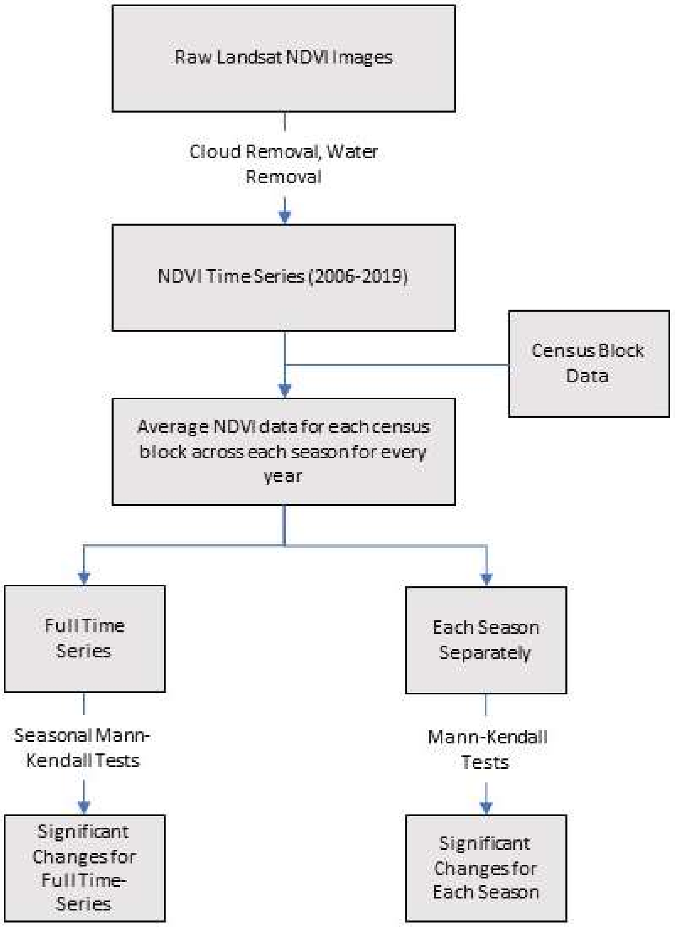

Supplementary Figure S2. Flow diagram of the methodology for detecting significant trends in greenness across the entire time series and individual seasons.
